# Aging with *Toxoplasma gondii* results in pathogen clearance, resolution of inflammation, and minimal consequences to learning and memory

**DOI:** 10.1038/s41598-020-64823-6

**Published:** 2020-05-14

**Authors:** Kathryn E. McGovern, Carla M. Cabral, Helena W. Morrison, Anita A. Koshy

**Affiliations:** 1 BIO5 Institute, University of Arizona, Tucson, Arizona United States; 2 College of Nursing, University of Arizona, Tucson, Arizona United States; 3 Department of Immunobiology, University of Arizona, College of Medicine, Tucson, AZ United States; 4 Department of Neurology, University of Arizona, Tucson, Arizona United States

**Keywords:** Parasitic infection, Parasite host response

## Abstract

Persistent inflammation has been identified as a contributor to aging-related neurodegenerative disorders such as Alzheimer’s disease. Normal aging, in the absence of dementia, also results in gradual cognitive decline and is thought to arise, in part, because of a chronic pro-inflammatory state in the brain. *Toxoplasma gondii* is an obligate intracellular parasite that establishes a persistent, asymptomatic infection of the central nervous system (CNS) accompanied by a pro-inflammatory immune response in many of its hosts, including humans and rodents. Several studies have suggested that the inflammation generated by certain strains of *T. gondii* infection can be neuroprotective in the context of a secondary insult like beta-amyloid accumulation or stroke. Given these neuroprotective studies, we hypothesized that a prolonged infection with *T. gondii* may protect against age-associated decline in cognition. To test this hypothesis, we infected young adult mice with either of two genetically distinct, persistent *T. gondii* strains (Prugniaud/type II/haplogroup 2 and CEP/type III/haplogroup 3) and monitored mouse weight, survival, and learning and memory over the ensuing 20 months. At the end of the study, we evaluated CNS inflammation and parasite burden in the surviving mice. We found that parasite infection had no impact on age-associated decline in learning and memory and that by 20 months post infection, in the surviving mice, we found no evidence of parasite DNA, cysts, or inflammation in the CNS. In addition, we found that mice infected with type III parasites, which are supposed to be less virulent than the type II parasites, had a lower rate of long-term survival. Collectively, these data indicate that *T. gondii* may not cause a life-long CNS infection. Rather, parasites are likely slowly cleared from the CNS and infection and parasite clearance neither positively nor negatively impacts learning and memory in aging.

## Introduction

An ongoing topic of discussion in the field of aging is the role of chronic inflammation in the brain, especially in the context of neurodegenerative disorders^1^. Although the mechanisms of inflammation-induced cognitive decline are not thoroughly understood, an increasing body of literature links chronic pro-inflammatory immune responses to accelerated memory deficits and neurodegeneration as observed in conditions like Alzheimer’s and Parkinson’s disease. With this work we sought to measure the impact of life-long inflammation in the CNS on normal aging by studying the long-term impact of infection with the neurotropic protozoan parasite, *Toxoplasma gondii*.

*T. gondii* is a ubiquitous obligate intracellular parasite that naturally infects most warm-blooded animals, including humans and rodents^2^. *T. gondii* has many genetically distinct strain types^3,4^ with the most studied strains being the canonical strains called type I, II, and III. The strains are categorized by genetic differences and acute virulence in mice. Type I strains are highly virulent (LD_100_ = 1 parasite) and kill mice before reaching the brain. Type III strains are avirulent (LD_50_~ 100,000), and type II strains fall in between the other two in terms of acute virulence (LD_50_~ 10,000 parasites). Most hosts become infected with *T. gondii* via the ingestion of contaminated food and water, after which the parasite causes an acute systemic infection. While the innate and adaptive immune response clears the parasite from many organs, in some organs the encysting parasite strains (e.g. type II and type III) switch from a fast-growing form to a slow-growing, encysted form. The encysted form is poorly recognized by the immune system, enabling a life-long, persistent infection^2,5,6^. In both humans and rodents, the brain is a major organ for encystment^7–9^.

In the initial phase of infection, when *T. gondii* is primarily present in the periphery, parasite exposure has been shown to be protective against subsequent bacterial^10,11^, viral^12–14^, fungal^15^, and parasitic^16–18^ challenge. In some cases, live parasites are not required to elicit this protective effect^11,14,18^. During early chronic brain infection in rodents, when there is measurable parasite antigen burden and high immune cell infiltration, infection has been linked to seizures^19,20^, altered fear responses^19,21,22^ and impairments in learning and memory^22–26^. However, infection has also been shown to be beneficial in the context of stroke^27^ and in three distinct models of Alzheimer’s disease^28–30^. In all three studies, infection protected the brain against plaque deposition, with one group reporting reduced amyloid-induced memory deficits^30^. Most of these studies have employed a type II *T. gondii* strain, though recently there has been an interest in the subtle differences that genetically distinct parasite strains may have on neuroprotection^28^, behavior^22^, and CNS immune responses^31^.

Given the beneficial effects of *T. gondii* on secondary CNS insults, we sought to determine if *T. gondii* infection could also protect against age-associated decline in memory and learning^32^, a much milder insult compared to the prior studies, but still associated with neuroinflammation^1^. In addition, to determine the *T. gondii* strain-specificity of any protective effect we found, we used both a type II strain and a type III strain. To that end, we infected young adult mice (3 months old) with either type II or type III parasites and monitored a number of parameters including memory until they reached 23 month of age, (20 months post infection (mpi)). Contrary to what is seen early during infection^33^, compared to type II–infected or uninfected mice, we found that type III–infected mice are chronically underweight and succumb to the infection more as they age. In the surviving mice, we found no effect of infection on learning and memory, as assessed by three different tests. Finally, contrary to prior work showing parasite persistence even at 22 mpi^2,6^ and our own work at 6 mpi^28^, at 20 mpi we found no evidence of persistent parasite infection in the CNS. Consistent with the lack of parasites in the CNS, by both flow cytometry and immunohistochemistry, we found no evidence of elevated neuroinflammation compared to age-matched, uninfected controls. Together these data indicate that infection with either of two genetically distinct, encysting *T. gondii* strains does not alter age-associated changes in cognition and suggest that parasites can be fully cleared from the CNS.

## Results

### Type III infection is more virulent to mice during life-span infection

**Figure 1:**
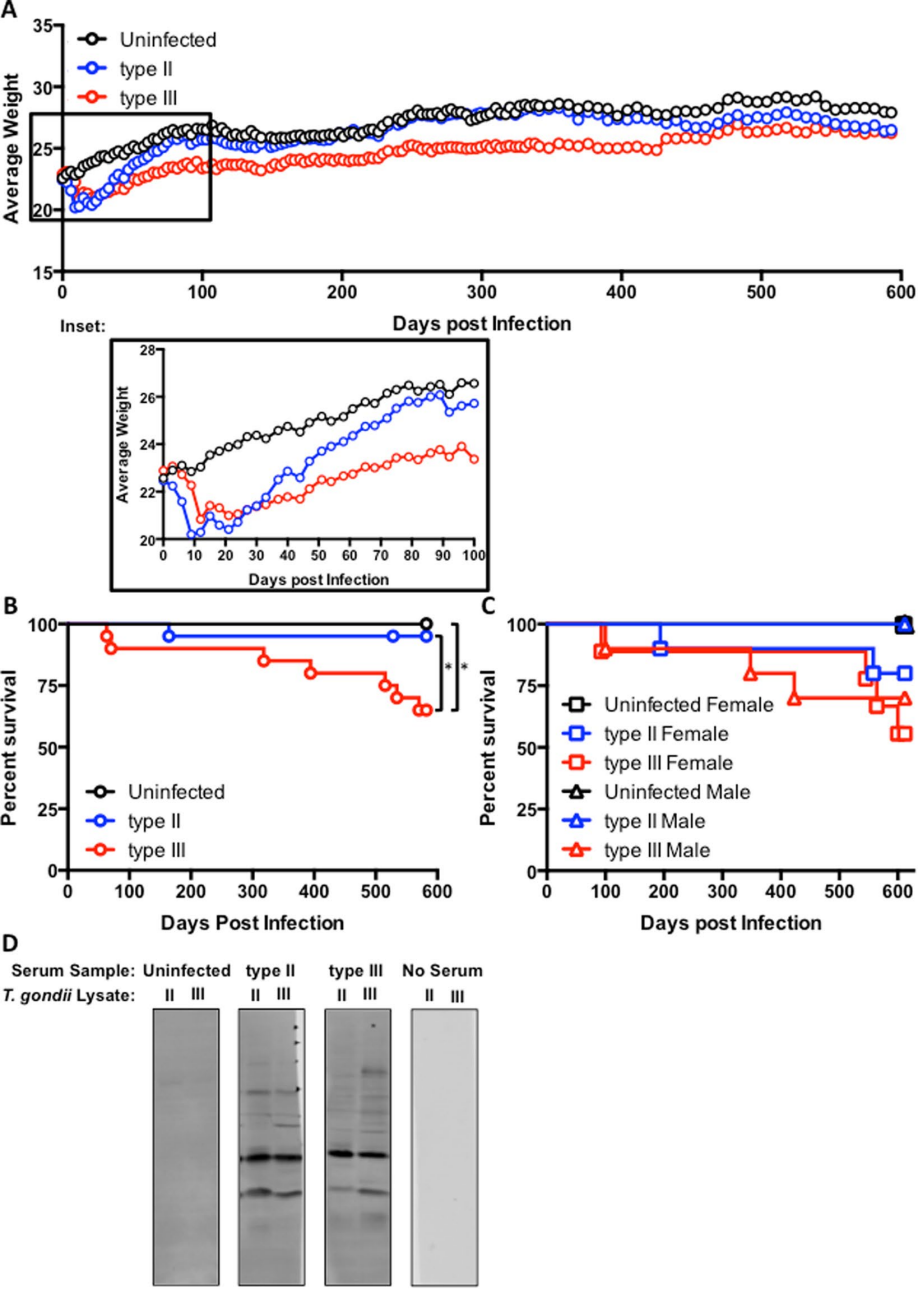
*More type III–infected mice succumb during life span infection*. Male and female mice were infected with either type II or type III parasite strains or inoculated with saline (total of 10 saline, 20 type II, and 20 type III, split evenly by gender) and monitored for changes in weight (**A**) and survival (**B** and **C**). (**B**) *p ≤ 0.05 (Uninfected vs. type III p = 0.046, type II vs. type II p = 0.050); Uninfected vs. type II p = 0.3365, Log-rank test. (**C**) Splitting the data by gender reduces power and statistical significance among groups is lost. (**D**) Representative western blots of type II and type III lysates probed with sera from individual surviving animals.

To test if *T. gondii* can protect against normal aging^32^ and determine if parasite strain type affects any potential protection, adult mice (three months old) were infected with either of the two cyst-forming canonical strains (type II Prugniaud and type III CEP) and monitored over the course of their natural life span. Given that we had previously noted that type III–infected mice had a far lower CNS parasite burden than type II–infected mice at 6 months post infection (mpi)^28^, we used a five-fold higher inoculation for mice infected with type III (50,000) vs type II (10,000) parasites. As expected, all infected mice lost weight during the acute phase of infection (Fig. 1A), with peak weight loss occurring at 10 days post infection (dpi) (Fig. 1A, inset), which is just as parasites are being cleared from the periphery and enter the CNS^34,35^. After this nadir, the mice regain weight, though type III–infected mice maintained a slightly lower average weight than type II–infected or uninfected animals (Fig. 1A) until over a year post infection. As expected for the inoculums we used, we had no mice die during the acute phase of disease (0–2 weeks post infection), and only one type II– and two type III–infected mice died in the first 6 mpi. After 6 mpi, the type II–infected mice had one more death through 20 mpi, while five type III–infected mice died between 10–20 mpi. (Fig. 1B). This increased death rate occurred in both male and female mice (Fig. 1C)^36,37^. Upon completion of the study, we confirmed that the remaining mice used for immunohistochemistry had been infected with *T. gondii* by using western blot-based serology testing (Fig. 1D). Collectively, these data suggest that infection with type III parasites has a greater impact on mouse weights and survival over the long term. In addition, this effect on long-term survival is not exclusively driven by the known susceptibility of female mice to *T. gondii* infection^36,37^.

### *Life-long* T. gondii *infection has no effect on learning and memory*

**Figure 2:**
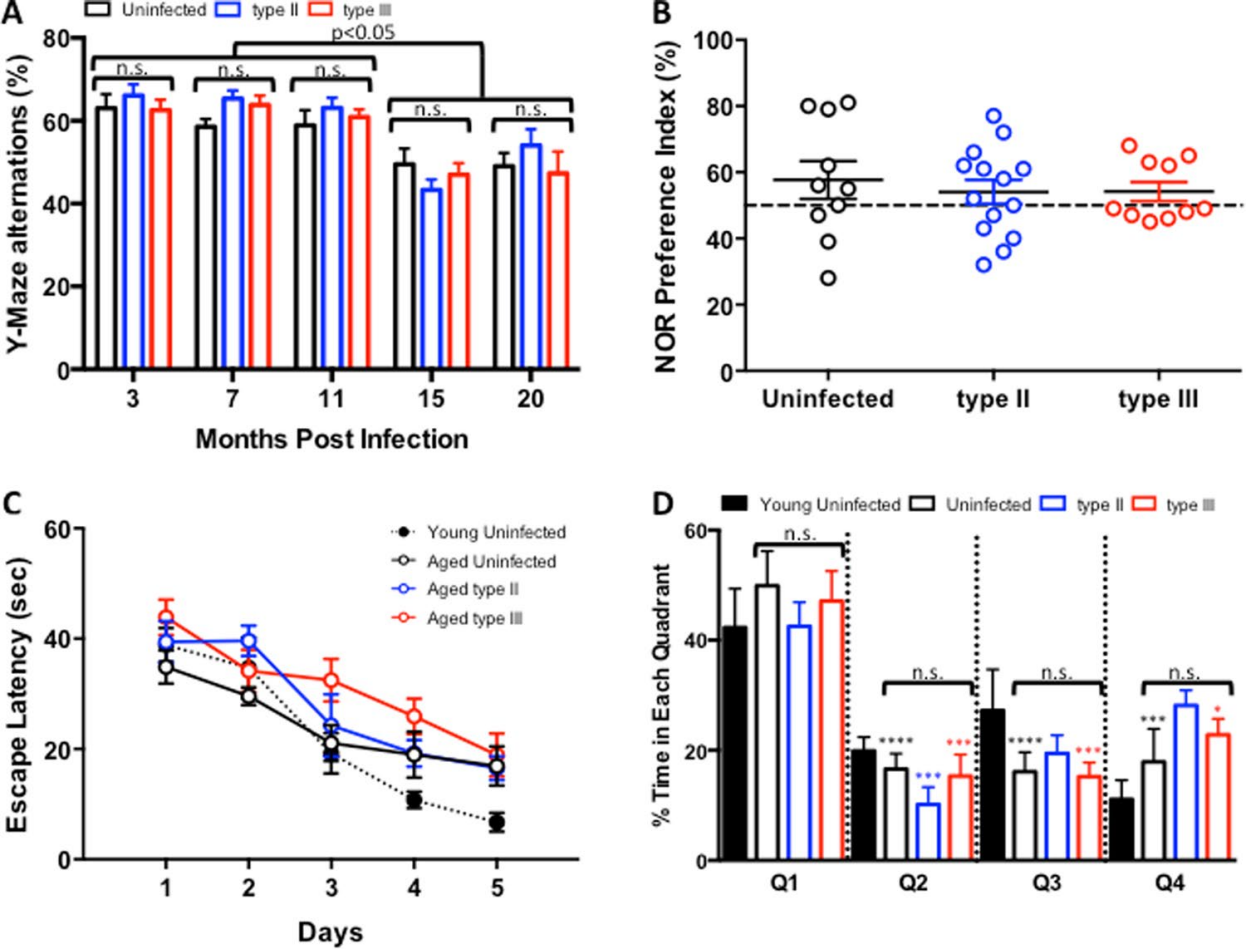
*T. gondii infection has no impact on age-associated decline in learning and memory*. Over the course of infection, infected and control animals were monitored for changes in memory and learning using 3 different tests: Y-maze, Novel Object Recognition, and Morris water maze. (**A**) Graph of the percentage of time mice in each group alternated which arm of a Y-maze was explored. For p values for individual comparisons, see Table S1. At 3 mpi, N = 10, 19, and 19 for uninfected, type II–, and type III–infected mice. At 7 and 11 mpi, N = 10, 18, and 18 for uninfected, type II–, and type III–infected mice. At 15 mpi, N = 10, 18, and 15 for uninfected, type II–, and type III–infected mice. At 20 mpi, N = 9, 17, and 11 for uninfected, type II–, and type III–infected mice. Two living mice (1 type II and 1 type III) were excluded for failing to explore the maze for more than 5 sec, and 1 type III mouse was excluded after developing a malocclusion. *p ≤ 0.05, **p ≤ 0.01 ***p ≤ 0.001, ****p ≤ 0.0001, Two-way ANOVA. (**B**) Quantification of how often mice in each group spent time exploring the novel object. Data displayed was collected at 20 mpi, where N = 10, 14, and 10 for uninfected, type II–, and type III–infected mice. Five living mice (3 type II and 2 type III mice) were excluded for failing to explore the maze for more than five seconds. (**C**) Quantification of the amount of time it took mice in each group to find the submerged platform in a water maze. The filled bubbles, dotted line is data generated by 8 young uninfected mice tested at a different time and has been superimposed upon the graph of the older mice to display the difference between young and old mice. Data displayed was collected at 20 mpi, where N = 9, 8, and 9 for aged uninfected, type II–, and type III–infected mice were tested. While 10 mice per group were randomly chosen for testing, one mouse died (one saline) and three mice were excluded due to blindness (two type II and one type III). (**D**) Quantification of the amount of time mice in each group spent in the quadrant of the water maze that contained the submerged platform. The filled bars are data generated by young uninfected mice tested at a different time and have been added to the graph of the older mice to display the difference between young and old mice. They have been excluded from statistical analysis. *p ≤ 0.05, **p ≤ 0.01 ***p ≤ 0.001, ****p ≤ 0.0001, One-way ANOVA. For p values for individual comparisons, see Supplemental Table 2. Number of mice per group is the same as in C. *A–D*. Bars, mean ± SEM. Significant differences do not change upon the inclusion of immobile or blind mice.

Even in the absence of injury, infection, or age-associated morbidities, normal aging is associated with cognitive decline in humans and rodents^32,38^. As type II strains of *T. gondii* have been shown to be neuroprotective during severe secondary challenges to the CNS^27–30^, we sought to determine if persistent *T. gondii* infection could slow or even prevent age-associated cognitive function. To assess this possibility, infected mice were subjected to three specific behavior tests prior to sacrifice. Over the course of infection, mice were placed in a Y-maze, a test that evaluates a rodent’s ability to remember which arm of the Y-maze it last entered and explore the “new” environment (the other arm) (Fig. 2A). Contrary to a previous report^39^, we found that the willingness of the mice to explore a new arm of the maze, as measured by the percentage of alterations, remained steady for all groups until the mice reached 15 months post infection (18 months of age). At that point, the percentage of alterations decreased significantly as compared to three months post infection. However, infection status and parasite strain did not significantly alter exploratory behavior (Fig. 2A). At both 15 and 20 mpi (18 and 23 months old respectively), mice were subjected to the Novel Object Recognition task (Fig. 2B, 20 mpi shown), which is used to evaluate short term/recognition memory^40,41^. Mice with intact recognition memory should spend more time exploring the novel object. On average, aged uninfected mice spent slightly more time exploring the novel object. Neither infection with type II nor type III parasite strains changed that behavior (Fig. 2B). Finally, to test both spatial and long-term memory, mice were subjected to the Morris Water Maze at 15 and 20 mpi (Fig. 2C and D, 20 mpi shown) and compared to young, uninfected mice tested in a separate session (Fig. 2C, filled data points with dotted line, overlaid; Fig. 2D, filled bars). Aged uninfected and infected mice, and young uninfected mice began the test with similar escape latencies, at approximately 40 sec (Fig. 2C). The ability of all groups to escape the maze improved over time. While young mice steadily and dramatically improved over 5 days to achieve an escape latency of approximately 7 sec, all aged groups plateaued at an escape latency of 20 sec. Neither infection status, nor parasite strain, significantly impacted their ability to escape the maze (Fig. 2C). Indeed, in the probe trial, all groups spent the majority of their time in the target quadrant (Q1) (Fig. 2D). Together, these data indicate *T. gondii* infection has no impact on age-related cognitive decline when assessed by these three different tests.

### Parasite are no longer detected in the CNS of aged mice

**Figure 3:**
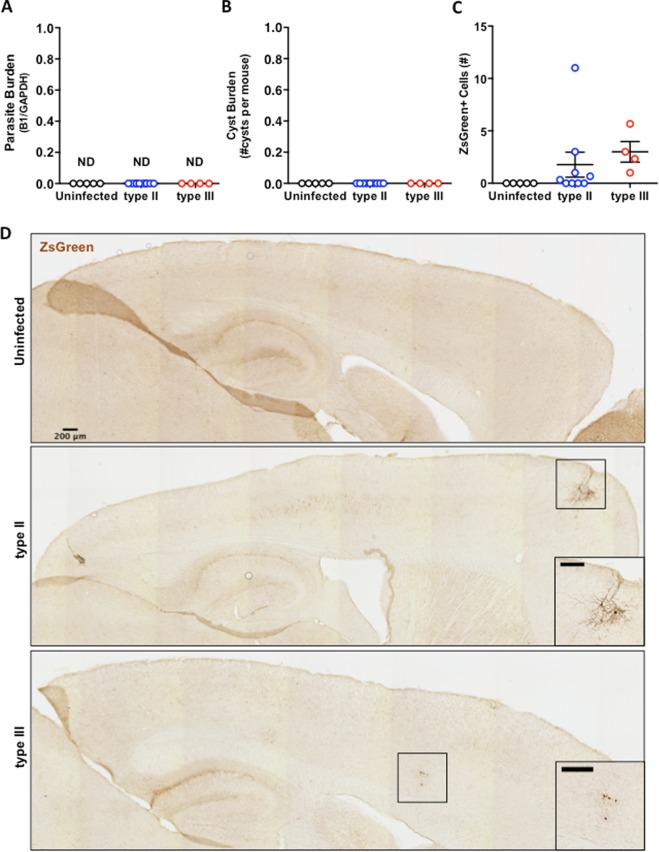
*Parasites are not detected in the brain at 20 mpi*. (**A**) Quantification of *T. gondii* burden in brain homogenate using RT-PCR for *T. gondii*-specific B1 gene and host GAPDH gene (housekeeping gene). (**B**) Quantification of *T. gondii* cyst burden in brain sections stained with Dolichos biflorous agglutinin, which stains the cyst wall. Stained sections were analyzed by epiflourescent microscopy to quantify DBA^+^, mCherry^+^ cysts. N = 12 sections/mouse. (**C**) Quantification of ZsGreen^+^ cells/tissue section in sections stained with anti-ZsGreen antibodies. Stained sections were analyzed by light microscopy and ZsGreen^+^ cell quantified manually. Numbers displayed represent the average number of ZsGreen^+^ cells counted on the three most medial brain sections per mouse (**D**) Representative images of stitched images of stained tissue from labeled groups. Insets show ZsGreen^+^ cells. Scale bar for both stiched images and insets equals 200 μm. *A–C*. N = 3 sections/mouse, 5 uninfected, 9 type II and 4 type III mice/group. Bars = mean ± SEM.

As *T. gondii* is thought to cause a life-long CNS infection^2,5,6^, we next sought to determine the CNS parasite burden in approximately half of our surviving mice (5 uninfected, 10 type II–, and 5 type III–inoculated mice), while the other half (4 saline, 8 type II–, and 7 type III–inoculated) were used for flow cytometry. To our surprise, we were unable to detect parasites in the brain either through RT-PCR using *T.gondii* gene-specific primers (Fig. 3A) or through staining tissue sections for cysts (Fig. 3B). Although we found no evidence of current infection, we had infected mice that permanently express the green fluorescent protein ZsGreen only after Cre-mediated recombination with type II and type III strains that inject a *Toxoplasma*:Cre fusion protein into host cells prior to host cell invasion^42,43^. This system allows us to identify and track CNS cells injected with parasite proteins, regardless of the current infection status of the cell^35^. To determine if we could find evidence of prior CNS infection, we examined tissue sections for CNS cells expressing ZsGreen. In uninfected mice, we found no cells expressing ZsGreen. Fifty percent of type II–infected mice and 100% of type III–infected mice had CNS cells that expressed ZsGreen (Fig. 3C). Consistent with prior work^35^ the ZsGreen^+^ cells were all neurons by morphology and were often observed in clusters (Fig. 3D). These data suggest that parasites enter the brain and are subsequently cleared. The very low number of ZsGreen^+^ neurons is consistent with prior data suggesting that these ZsGreen^+^ neurons peak in the first several weeks post-infection and then decrease in number over time^35^.

### At 20 mpi, parasite-exposed mice have the same number of CNS T cells as aged uninfected mice

**Figure 4:**
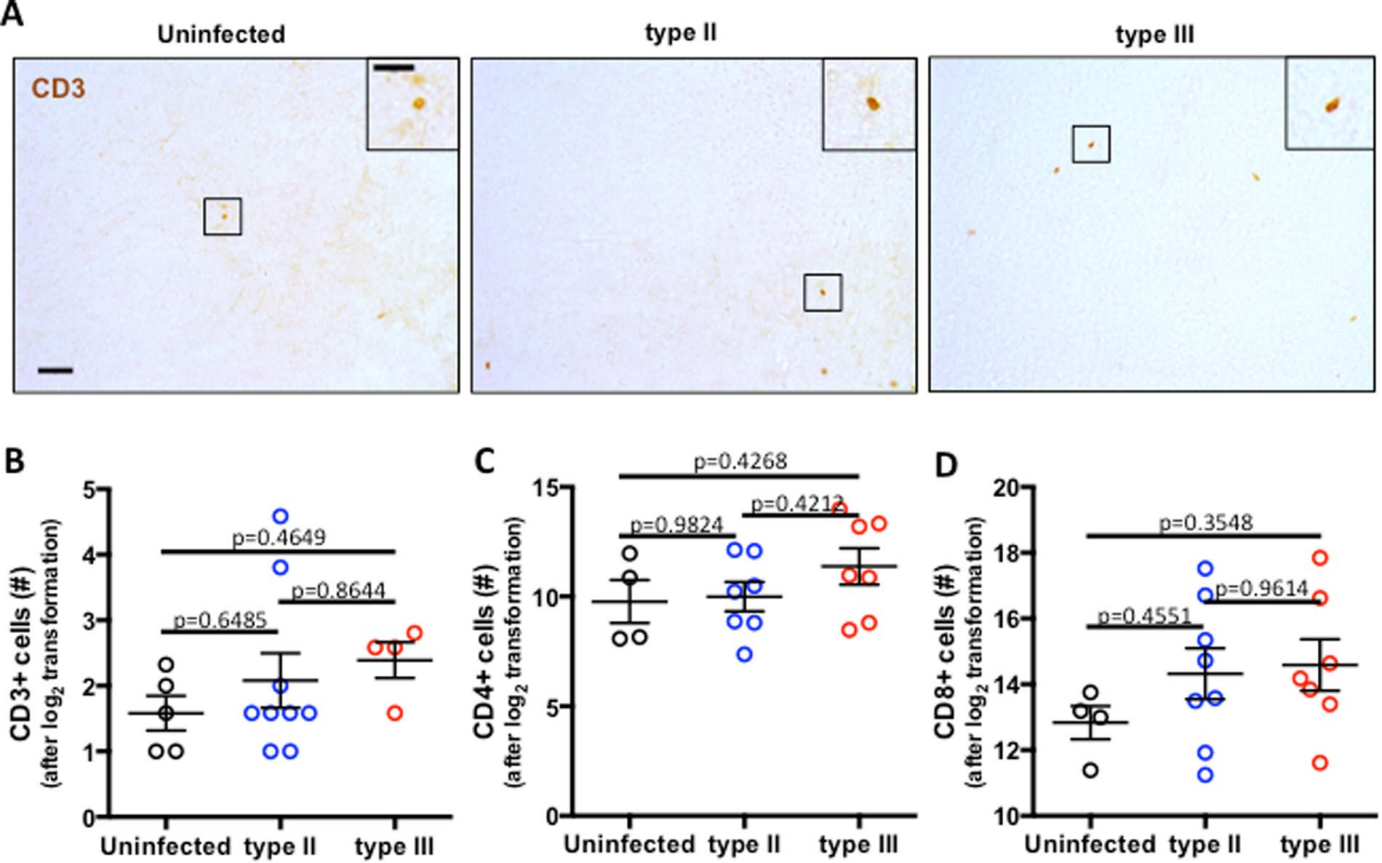
*T cell numbers in brains of parasite-exposed mice are equal to uninfected mice*. (**A**) Representative images of CD3^+^ cells (T cells) in brain sections stained with anti-CD3 antibodies. Full image scale bar = 50 μm, inset scale bar = 25 μm. (**B**) Quantification of CD3^+^ cells/field of view/mouse. N = 12 fields of view/section, 3 sections/mouse averaged together, 5 uninfected, 9 type II, and 4 type III mice. (**C**) Quantification of the number of CD4^+^ T cells (CD3^+^CD4^+^) isolated from the brain and assessed by flow cytometry. (**D**) Quantification of the number of CD8^+^ T cells (CD3^+^CD4^-^) isolated from the brain and assessed by flow cytometry. *C, D*. N = 4 uninfected, 8 type II, and 7 type III mice/group. *B-D*, raw data were log_2_ transformed prior to analysis and graphing. *B–D*, Bars = mean ± SEM.

T cell infiltration into the CNS is critical for keeping parasite replication under control^44^. During peak antigen burden, millions of T cells can be found in the brain parenchyma^31,45^ and those numbers remain elevated even at 6 months post infection^28^. Given the lack of evidence for active infection, we sought to determine how many infiltrating T cells remained by analyzing tissue sections stained for CD3, a surface antigen common to all T cells (Fig. 4A). T cell infiltration was found to be low, with considerably fewer cells found in the brain than historically identified at the early stages of chronic infection^45^. When quantified (Fig. 4B), T cell infiltration was similar to baseline infiltration found in aged, uninfected mice. To complement the immunohistochemical analysis, we used flow cytometry to determine if differences in the make-up of T cell subpopulations were present. However, the numbers of both CD4^+^ (Fig. 4C) and CD8^+^ (Fig. 4D) T cells in both type II– and type III–infected animals were indistinguishable from those found in uninfected aged animals. These data are consistent with previous reports indicating that to be retained in the brain, a T cell’s cognate antigen must be present^46^ and therefore are supportive of the data in Fig. 3 that parasites are no longer present in the brain. Together these data indicate that T cells are no longer drawn to or retained in the brain once parasites are cleared.

### At 20 mpi, parasite-exposed mice show the same myeloid cell infiltration/ activation as aged uninfected mice

**Figure 5:**
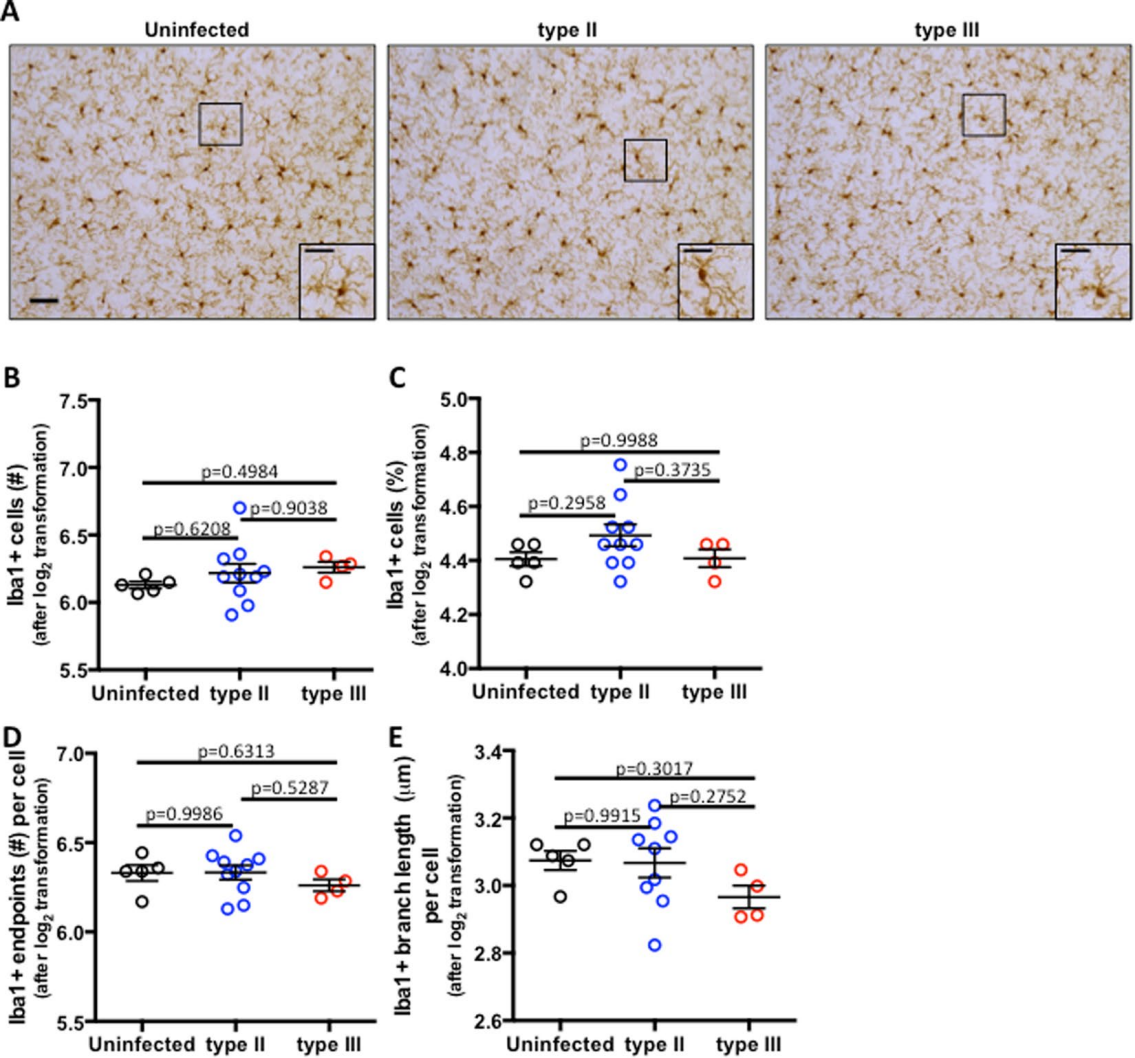
*Myeloid lineage cell numbers and morphology return to baseline*. (**A**) Representative images of Iba1^+^ cells (microglia and infiltrating macrophages) in fixed tissue sections stained with anti-Iba1 antibodies. Full image scale bar = 50 μm, inset scale bar = 25 μm. (**B**) Quantification of the number of Iba1^+^ cell bodies/field of view/mouse. (**C**) As in (*B*) except quantifying the percent of area staining for Iba1. *D, E*. Individual myeloid cells were evaluated for the number of branch endpoints per cell (**D**) and the average branch length per cell (**E**). N = 12 fields of view/section, 3 sections/mouse averaged together, 5 uninfected, 9 type II and 4 type III mice/group. *B–E*, raw data were log_2_ transformed prior to analysis and graphing. *B–E*, Bars = mean ± SEM.

Myeloid lineage cells also play a role in keeping parasite replication in check^47,48^. These include resident microglia that proliferate in response to infection, and monocytes that infiltrate from the periphery into the CNS and become macrophages. Myeloid cells contribute to cytokine and chemokine production in the inflamed brain^49,50^ and participate in parasite killing^48,51–53^. In the presence of parasites, staining for Iba1, a marker that labels both resident microglia and infiltrating macrophages, increases considerably compared to naïve animals and can vary dramatically between strain type even at 6 mpi, when the parasite burden is considerably lower than early in CNS infection^2,28,31^. To evaluate CNS microglia/macrophages in aged mice, tissue sections were stained for Iba1 (Fig. 5A) and analyzed for cell number (Fig. 5B) and percentage of positively stained tissue (Fig. 5C). Consistent with our T cell data, infected and uninfected sections showed similar numbers of Iba1^+^ cells and levels of staining, indicating that myeloid cell infiltration, proliferation, and activation resolves in the absence of parasite antigen.

To further characterize microglial/infiltrating-macrophage activation, we evaluated the morphology of Iba1^+^ cells in histological sections. Such analysis is important because brain Iba1^+^ cells, which in uninfected mice are primarily microglia, are highly arborized cells with dynamic processes^54^ that change morphology in response to disease and injury^54–57^. For example, highly ramified Iba1^+^ cells are associated with microglia surveillance functions and maintenance of neural networks^55–57^ whereas de-ramified cells are associated with post-injury or disease responses such as debris/pathogen phagocytosis^58^. Thus, Iba1^+^ cell morphology is thought to be coupled to function; however, the precise relationship between form and function has yet to be fully determined. To look for subtle differences in myeloid cell morphology, which might suggest differences in activation states, we analyzed Iba1^+^ cell morphology in tissue sections using a standardized ImageJ-assisted skeleton analysis method that allows quantification of the number of endpoints per cell and the average branch length per cell, which are a more objective and precise measurement of morphology^59,60^. Using this methodology, we found that neither endpoints per cell nor branch length per cell were statistically different between infected or uninfected mice, regardless of infecting strain. Collectively, these data are consistent with myeloid inflammation resolving after pathogen clearance, leaving minimal changes in myeloid lineage cell morphology.

## Discussion

Here we sought to understand how a long-term infection with *T. gondii*, a parasite thought to establish a life-long infection within the CNS, affects the natural cognitive decline associated with aging. Our data strongly suggest that infection with either of two canonical encysting strains of *T. gondii* does not impact age-related cognitive decline of memory or learning. In addition, and unexpectedly, we found that infection with a type III strain that is highly avirulent during acute infection shows a higher-level of lethality during chronic infection. In the surviving mice infected with either *T. gondii* strain, we found no evidence for persistent parasite infection at 20 mpi. Consistent with a lack of parasite antigen in the CNS, *T. gondii*-inoculated mice showed no evidence of persistent inflammation in the brain. Collectively, these findings suggest that *T. gondii* is cleared from the brain, albeit slowly.

The lack of any effect on memory and learning during aging is of interest because many studies– using a range of rodents– have found that *T. gondii*-infected hosts show a range of behavioral abnormalities, including in memory and learning^19,21–26,61^. One clear difference between our work and the prior studies is that we performed the majority of our testing at 20 mpi, while most of the prior work has assessed behavior from three to eight weeks following infection, a time frame in which the CNS would be expected to have high levels of parasites/parasite antigen and inflammation. In fact, the only other study that conducted memory and learning studies at later time points (6 mpi, 9 months of age) also reported no change in memory and learning compared to equivalently aged uninfected mice^30^. Thus, one possible explanation for these discrepancies would be that the effect on learning seen during “early” chronic infection may relate to parasite burden and the corresponding neuroinflammation, leading to recovery of normal behavior as the parasite burden and inflammation decrease. Of note, mounting evidence suggests the level of neuroinflammation, not parasite burden, is responsible for *T. gondii*-induced behavioral changes^22,26,61,62^.

A second interesting finding from our study is the increased rate of death in aging mice inoculated with type III parasites. Type III strains are considered highly avirulent in mice while type II strains are considered more virulent^33^. These differences in virulence were defined during acute infection and have rarely been examined past 2–4 mpi^50^. Consistent with the known differences in acute virulence, compared to type III–infected mice, type II–infected mice lost weight slightly earlier and faster during systemic infection (Fig. 1A) despite being inoculated with 10-fold fewer parasites. However, at later points in chronic infection, the type II–infectedmice recovered, consistently reaching the weights of uninfected mice by ~ 150 dpi (Fig. 1A). On the other hand, type III–infectedmice, which had less initial weight loss (Fig. 1A), did notably worse over the long-term (Fig. 1B,C). As the mice that died did not undergo necropsy and the survivors appeared equivalent to the uninfected mice in terms of CNS inflammation and parasite burden, it is unclear what drove the higher age-associated mortality rate in type III–inoculated mice. We speculate that this increased mortality may be secondary to a prolonged and more pro-inflammatory CNS response in type III–infected mice because our recent work suggests that at 3 wpi type III–infected mice have more T cells and macrophages in the brain compared to type II–infected mice with equivalent inoculums^31^. In addition, in an Alzheimer’s disease mouse model, at 6 mpi, type III–infected mice had an equivalent inflammatory burden to type II–infected mice even though they had 100-fold decrease in parasite burden as assessed by qPCR for a *T. gondii*-specific gene^28^. These findings suggest that the difference in the immune response to type II or type III– infection^31^ could underlie the differences we found in age-associated mortality. Another possibility is that the mortality difference is not *T. gondii* strain-specific but, rather, secondary to the different initial inoculums. The use of a five-fold higher inoculum for the type III strain may have led to an increased parasite burden in the early part of acute and chronic infection, which in turn could provoke an increased inflammatory response that affected long-term mortality. Consistent with the possibility that the higher inoculum led to an increase in parasite burden 100% of the III–Cre infected mice had ZsGreen^+^ neurons compared to only 50% of the II–Cre infected mice. Ultimately, our results challenge the notion that type III parasites are completely avirulent, therefore how we define the virulence of a parasite strain may also need to be reconsidered.

The most surprising result from this study is the seemingly complete clearance of parasites from the brain and the resolution of inflammation. Prior reports determined that *T. gondii* infection persists in the brain throughout the lifetime of a murine host^2,5,6^. While we expected there to be differences in parasite burden between strains, as is evident six months following infection^28^, we did not expect a lack of parasites in the brain. This difference from the original reports, the only other studies to address brain cyst burden over the lifetime of a mouse, maybe secondary to differences in the *T. gondii* and mouse strains used. The original reports used either SRRA^2,6^ or Me49^5^, type II strains that are known to cause a high CNS cyst burden and significant pathology in the murine model, while we used Prugniaud, a type II strain that leads to lower cyst burden and less pathology, and CEP, a type III strain. Further, the original reports used outbred mice, while we used in inbred C57Bl/6 line. Thus, differences in the peak cyst burden or strain-specific likelihood of reactivating and re-infecting new CNS host cells, in addition to the polarization of host immune cells or genetic variation that affects intrinsic clearance mechanisms, may affect the rate of clearance of parasites from the CNS. We are confident that this difference is not because our mice were not infected or the parasites did not make it to the brain, as all *T. gondii* inoculated mice lost weight during acute infection (Fig. 1A); all tested surviving mice were seropositive (Fig. 1D); and most surviving infected mice showed evidence of neurons injected with *T. gondii* effector proteins (Fig. 3C,D)^42,43^. While it is possible that some CNS cells spontaneously expressed ZsGreen (i.e. not secondary to parasite-triggered Cre-mediated recombination), ZsGreen^+^ neurons were not found in any of the uninfected mice.

In the setting of apparent complete clearance of parasites from the brain, CNS inflammation, as measured by T cell infiltration and microglia/macrophage activation/infiltration, also resolves (Figs. 4 and 5). This resolution is consistent with the brain’s status as an immune privileged, but not immune isolated, site. Without a source of cognate antigen or chemokines/cytokines, there are no cues for infiltrating effector cells to remain in the brain or for microglial activation to continue^46^. We conclude that the natural mechanisms in place to combat the parasite in the brain are effective, albeit slow. This slow response may be another level of regulation that ensures precision in a site that is so sensitive to immune-mediated pathology. The existence of an effective clearance mechanism could explain why 50% of *T.gondii*-seropositive AIDS patients never experience reactivation^63,64^. Future work will need to define the mechanism(s) that lead to parasite clearance and determine whether they can be enhanced to hasten clearance and even applied to other pathologic CNS infections.

Although it is not possible to know at what time point individual neurons have interacted with a parasite, the presence of residual *T. gondii*-injected neurons (Fig. 3) in the context of apparent parasite clearance opens intriguing possibilities about neuron-*T. gondii* interactions. If these neurons were injected with parasite protein (either with or without subsequent invasion) during early CNS infection, their survival would suggest that not all *T. gondii*-injected neurons are cleared from the CNS. The lack of clearance over the long-term would imply that these neurons are functional, as non-functional neurons are normally cleared by microglia^65,66^. Alternatively, if parasitic cysts continually rupture, individual parasites may interact with and invade new neurons continually throughout the host’s lifetime. Consequently, these surviving ZsGreen^+^ neurons may have been injected by parasites just prior to the end of the study and, thus, were not yet cleared. New tools will need to be developed to address these questions of parasite-neuron-immune system interactions in the brain.

In summary, we report that *T. gondii* does not impact age-associated cognitive decline in mice and that by 20 mpi, for two encysting strains of *T. gondii*, parasites are cleared from the CNS. In addition, the corresponding CNS inflammation also resolves. These findings may imply that learning defects caused by the parasite are temporary or dependent on parasite strain. We hope other investigators will begin to examine the permanency of infection-related behavior and how those changes might tie to parasite persistence or inflammation. In addition, given that with this study we cannot answer why the type III infection led to higher mortality rates in aging mice or the kinetics of parasite clearance and the resolution of neuroinflammation, our future work will focus on addressing these issues. Ultimately, this work highlights why studies that look at the full cycle of chronic infection offer different insights than those that come from focusing exclusively on short-term infection.

## Methods

### Mice

Cre reporter mice (6.Cg-Gt(ROSA)26Sort^m6(CAG-ZsGreen1)Hze^/J) on a C57Bl/6 background^67^ were purchased from Jackson Laboratories, bred, and aged at the BIO5 Institute’s animal facility at the University of Arizona. All mouse studies and care were carried out in accordance of protocols approved by the University of Arizona Institutional Animal Care and Use Committee.

### *T. gondii* maintenance and mouse infection

Parasites engineered to express mCherry and Cre recombinase^43^ were maintained in culture by serial passage in human foreskin fibroblasts (provided by John Boothroyd, Stanford University, Stanford, CA) in complete DMEM (Gibco) (10% FBS, 2 mM Glutagro, and 1% penicillin/ streptomycin) for up to 40 passages. After 40 passages, parasites that have been recently passed through CBA/J mice are thawed. For inoculation, parasites were syringe-released by repeated passage through a 25 G needle followed by passage through a 5μm nylon syringe filter; then pelleted at 3000 rpm for 10 min at 4 °C; resuspended in PBS; and counted. At 3 months of age, male and female mice were injected intraperitoneally with an inoculum of 10,000 type II (Pru-mCherry-toxofilin-Cre, 10 females, 10 males), 50,000 type III (CEP-mCherry-toxofilin-Cre, 10 females, 10 males) parasites, or vehicle (USP Grade sterile PBS) (5 females, 5 males). The passage number for Pru and CEP were equivalent. Mouse weights and survival were monitored over the 20-month course of infection. Two mice (female, saline and type III) were excluded from the study after developing a malocclusion at 20 and 14mpi (respectively). These mice were excluded as malocclusions spontaneously occur and are not associated with age or *T. gondii* infection.

### Y-Maze testing

To measure spontaneous alteration behavior, mice were placed in one arm of a Y-shaped maze composed of beige plastic with the following measurements: three arms set 120° apart with dimensions of 7.5 cm width × 37.0 cm length × 12.5 cm height. At 3, 7, 11, 15, and 20 months post infection, each mouse was allowed to freely explore the three arms for 5 minutes. Entry into a new arm of the maze was recorded when all four limbs of the mouse were within an arm. Mouse behavior was recorded and quantified using ANY-maze behavioral video tracking software (Stoelting). Data displayed are derived from the following mouse numbers: at 3 months post infection, N = 10, 19, and 19 for uninfected, type II–, and type III–infected mice (1 type II failed to explore the maze and 1 type III died prior to 3 mpi); at 7 and 11 months, N = 10, 18, and 18 for uninfected, type II–, and type III–infected mice (1 type II died between 3–7 mpi, 1 type II failed to explore the maze, and 1 type III died between 3–7 mpi); at 15 months, N = 10, 18, and 15 for uninfected, type II–, and type III–infected mice (2 type III mice died between 11–15 mpi, 1 mouse (female, type III) was excluded from the study after developing a malocclusion 14mpi); at 20 months, N = 9, 17, and 11 for uninfected, type II–, and type III–infected mice (1 saline died between 15–18mpi, 1 type III was excluded for failing to explore the maze, and 3 type III mouse died between 15–20 mpi). Mice that explored the maze for less than 5 seconds were excluded from analysis. Significant differences do not change upon the inclusion of immobile mice.

### Novel object recognition

At 15 and 20 months post infection, mice were habituated to the empty testing arena, a white plastic box (38 cm width × 48 cm length × 20 cm height), over two days with 15 min of exposure per day. The next day, each mouse was placed in the arena with two identical objects located at opposite corners of the arena. Mice were allowed to explore the objects for 5 minutes. Four hours later, one object in the arena was replaced with a new object and each mouse was allowed to explore the arena again. Different objects were used for each timepoint. Object exploration was defined by the time the mouse’s nose came within 2 cm of the object. Mouse behavior within the arena was recorded using ANY-maze behavioral video tracking software and the percentage of recognition was calculated using the following equation: [(Novel Object Time)/(Novel Object Time + Familiar Object Time)] × 100. Data displayed were collected at 20 months post infection, where N = 10, 14, and 10 for uninfected, type II–, and type III–infected mice. Mice that explored either object for less than 5 seconds (3 type II and 2 type III) were excluded from analysis. Of note, 1 type II mouse died between Y-maze testing and NOR testing. Significant differences do not change upon the inclusion of immobile mice.

### Water maze testing

A circular pool (diameter = 1.5 m, depth = 0.5 m) was filled with water maintained at 21 °C. The water was made opaque by mixing in non-toxic white paint powder (Jack Richeson and Co. Inc., Kimberly, WI, USA). Large visual cues were hung around the room that housed the tank to aid the mice to navigate while swimming in the tank. At 15 and 20 months post infection, each mouse was allowed to swim for 60 s and the time spent to reach a 15 cm platform submerged 2 cm below the water surface and therefore hidden from view (escape latency) was recorded in each trial during the training period. Each mouse had 6 training trials per day over a 5-day period. In each trial, mice were introduced to the water at randomized points around the tank. Mice that successfully found the platform were allowed to stay on the platform for 20 sec., whereas those that failed to locate the platform within the defined time frame were placed on the platform for 20 s by the experimenter. Two probe trials were conducted by removing the platform on the 6th day and two days later. Then, two visual trials were conducted on days 7 and 8 by placing the platform above the water line and marking it with a flag to confirm that the mice were able to see. Mouse behavior within the arena was recorded and analyzed using ANY-maze behavioral video tracking software. In order to test a representative number of mice from each group within one day for consecutive days, when possible, ten mice from each group were tested at 15 and 20 months post infection. Data displayed were collected at 20 months post infection, with N = 9, 8, and 9 for aged uninfected, type II–, or type III–infected mice respectively. Mice identified as blind by visual testing (2 type II and 1 type III) were excluded from analysis. The results from 8 young uninfected mice are also overlaid but excluded from statistical analysis because these data were collected separately. Significant differences do not change upon the inclusion of blind mice.

### Tissue processing

At 20 months post infection, mice were anesthetized with a 24 mg/mL:4.8 mg/mL ketamine:xylazine cocktail, a blood sample was taken, then mice were perfused with 0.9% saline. The cohort was then divided such that 5 saline-, 10 type II–-, and 5 type III–-inoculated mice were used for serology, tissue sections and parasite burden and 4 saline-, 8 type II–-, and 7 type III–-inoculated mice were processed for flow cytometry. For sectioning, brains were harvested and the left hemispheres drop fixed in 4% PFA for 24 hours followed embedding in 30% sucrose. Forty μm thick sagittal sections were sliced (and distributed across 12 tubes such that each section is 480 μm apart) using a freezing microtome (Microm HM 430). Sections were stored in cryoprotective media (0.05 M sodium phosphate buffer with 30% glycerol and 30% ethylene glycol) until stained. For parasite burden by RT-PCR, the right hemisphere was bisected coronally into two equal parts, placed in individual tubes, flash frozen, and stored at −80 °C until further processed.

### Serology testing

Serum from blood samples taken from each mouse were stored at −80 °C until tested for the presence of anti-*T. gondii* antibodies. Protein lysate from 5 × 10^6^ parasites of each strain were run on standard 10% acrylamide SDS-PAGE gels and transferred to PVDF membrane. The PVDF membranes were then cut into strips, blocked with 5% powdered milk, and each strip individually incubated with serum from each infected mouse at a dilution of 1:50. After washing, all strips were incubated in donkey anti-mouse secondary antibody conjugated with DyLight 800 (Life Technologies), washed, and then imaged on an Odyssey CLx (Li-COR).

### Flow cytometry

Brain mononuclear cell (BMNC) suspensions were prepared by mincing whole brain tissue with a razor blade and passaging 5 times through an 18-gauge needle. The resulting suspension was filtered through a 70 μm cell strainer, washed with cRPMI (10% FBS, 1%penicillin/streptomycin, 1% glutamine, 1% sodium pyruvate, 1% nonessential amino acids, and 0.1% beta-mercaptoethanol), and centrifuged at 1200 rpm for 10 min at 4 °C. The pellet was resuspended in 60% percoll and overlaid with 30% percoll. The percoll gradient was centrifuged at 2000 rpm for 25 min at room temperature. The BMNCs were harvested from the percoll interface and washed with cRPMI prior to counting, staining, and analysis. Single cell suspensions from the spleen and cervical draining lymph node were prepared by passing each tissue through a 40 μm cell strainer, washing with cRPMI, and centrifuging at 1200 rpm for 10 min at 4 °C. Red blood cells were lysed in 0.86% ammonium chloride solution and washed away with cRPMI. Single cell suspensions were counted then stained with the following antibodies: CD3-AmCyan (BD Bioscience), CD4-PECy7 (ebioscience). Samples were collected on the BD LSR II and analyzed using FlowJo (Tree Star Inc.).

### Immunohistchemistry

Free floating tissue sections were stained as previously described^28^. For immunohistochemistry, the following primary antibodies were used: polyclonal rabbit anti-Iba1 (019–19741, Wako Pure Chemical Industries, Ltd., 1:3000); monoclonal hamster anti-mouse CD3ε 500A2 (550277, BD Pharmingen, 1:500), rabbit anti-ZsGreen antibody (632474, Clontech, 1:5,000). Biotinylated goat anti-rabbit (BA-1000, Vector Laboratories, 1:500) and biotinylated goat anti-hamster (BA-9100, Vector Laboratories, 1:500) were used as secondary antibodies, as appropriate. For cyst detection, tissue sections were stained with biotinylated Dolichos biflorous agglutinin (1:500) followed by AlexaFlour 405-conjugated streptavidin (1:2000). Following staining, tissue sections were mounted onto slides for further analysis.

### Image analysis

All immunohistochemistry images were obtained a Leica DMI6000 motorized inverted microscope. For CD3 and Iba1 quantification, 12 fields of view from 3 sections per mouse were imaged across the cortex. The number of CD3^+^ and Iba1^+^ cells were counted manually. The percent area of Iba1^+^ cells in the cortex was calculated using thresholding with FIJI software^68^. Microglial morphology was analyzed as described previously^59^. Using this method we report ramification as two measures: number of endpoints per cell and summed process length per cell. Image analysis was carried out on the entire field of view. Identification of ZsGreen-expressing neurons was done by imaging and stitching the entire cortex from the 3 most medial sections per mouse and manually counting for ZsGreen^+^ cells. Cyst quantification was done as previously described^31^. In brief, brain sections (12 sections per mouse spanning across a hemisphere) were evaluated on an epifluorescent microscope (EVOS) and the number of Dolichos^+^, mCherry^+^ cysts were counted manually. One type III–infected mouse was excluded as an outlier. Statistical significance is not changed upon the inclusion of the outlier.

### qPCR for parasite burden

Genomic DNA was isolated from the rostral quarter of frozen brain tissue samples using a DNeasy Blood & Tissue Kit (69504, Qiagen) following manufacturer instructions. Amplification of the *T. gondii* B1 gene was performed using the Eppendorf Mastercycler ep realplex 2.2 system with the following reaction: 50 °C for 2 min, 95 °C for 2 min then 40 cycles of 15 sec at 95 °C and 1 min at 60 °C followed by a melting curve analysis^51^. The master mix was comprised of 10 μl SYBRGreen PCR Master Mix (4472908, Applied Biosystems), 0.5 μl of each primer (forward 5′ - AGG TCG GTG TGA ACG GAT TTG − 3′; reverse 5′ - AGC GTT CGT GGT CAA CTA TCG ATT G − 3′) at a concentration of 10 μM/μL, 4 μL of DNA at a concentration of 10 ng/μL and 5 μL of sterile nuclease free water. Standards, samples and negative controls were analyzed in triplicate for each run. In uninfected mice, the lowest average CT value was 37, therefore, values ≥37 were considered below the level of detection.

### Statistical analysis

All statistical analysis was performed using Prism software (Graph Pad Software, Inc.). As all comparisons were between multiple groups, one-way ANOVAs were performed to determine significance. One type III–infected mouse was excluded as an outlier during image analysis. To improve distributional characteristics between analyzed groups, data for cell counts were log_2_ transformed prior to analysis and graphing. Transformed data is indicated in the figure legend.

## Supplementary information

Aging with Toxoplasma gondii results in pathogen clearance, resolution of inflammation, and minimal consequences to learning and memory.

## Data Availability

The data generated during the current study are available from the corresponding author upon reasonable request.
