# Supplementary material for: Aging with Toxoplasma gondii results in pathogen clearance, resolution of inflammation, and minimal consequences to learning and memory
Source: Sci Rep. 2020 May 14;10:7979. doi: 10.1038/s41598-020-64823-6 (PMC7224383; doi:10.1038/s41598-020-64823-6)
Supplement: Supplementary file 1 — Aging with Toxoplasma gondii results in pathogen clearance, resolution of inflammation, and minimal consequences to learning and memory. [file 41598_2020_64823_MOESM1_ESM.docx]

Aging with *Toxoplasma gondii* results in pathogen clearance, resolution of inflammation, and minimal consequences to learning and memory

Kathryn E. McGovern^1^, Carla M. Cabral^1^, Helena W. Morrison^2^, and Anita A. Koshy^1,3,4^.

^1^BIO5 Institute, University of Arizona, Tucson, Arizona, United States.

^2^College of Nursing, University of Arizona, Tucson, Arizona, United States.

^3^Department of Immunobiology, University of Arizona, College of Medicine, Tucson, AZ, United States.

^4^Department of Neurology, University of Arizona, Tucson, Arizona, United States.

Electronic address: [akoshy@email.arizona.edu](mailto:akoshy@email.arizona.edu).


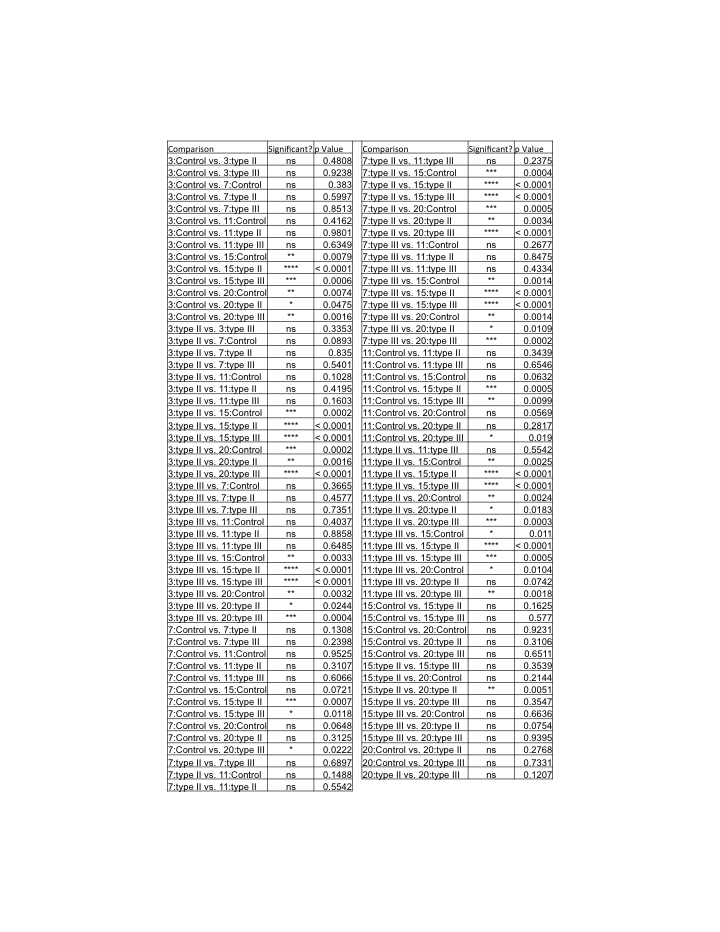


Table S1: List of individual comparisons and resulting p values for the Y maze behavioral study.


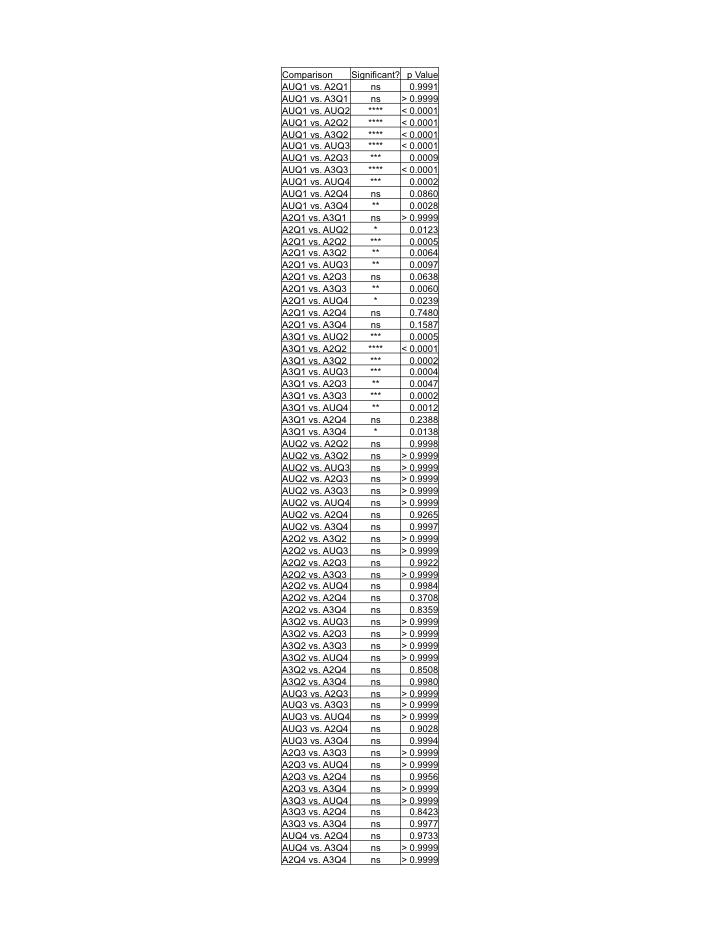


Table S2: List of individual comparisons and resulting p values for the water maze behavioral study.


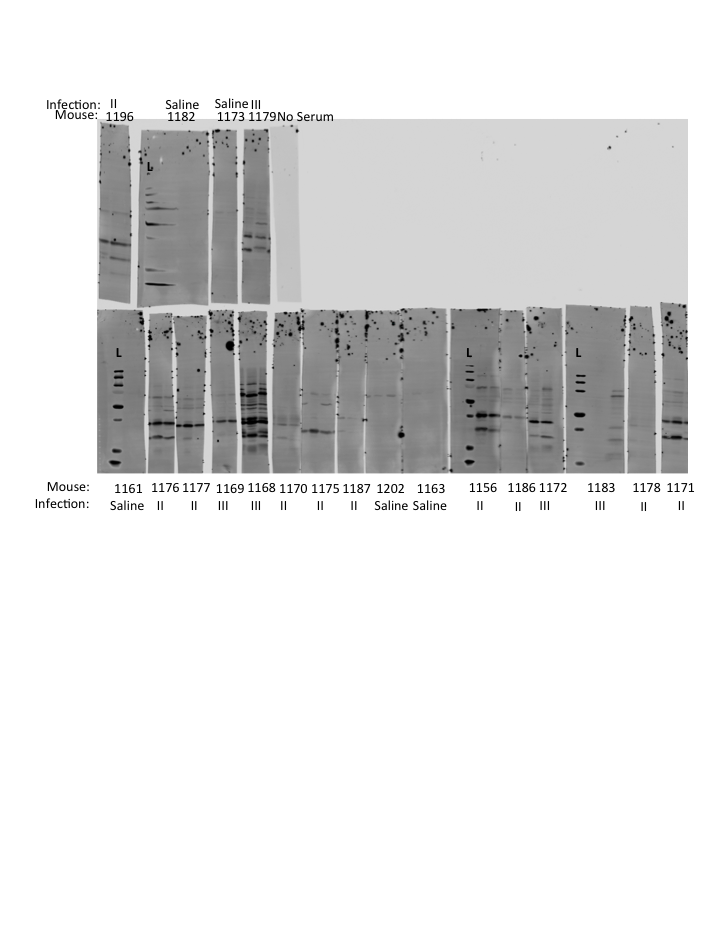


Figure S1: Uncropped western blot strips for *T. gondii* seropositivity.


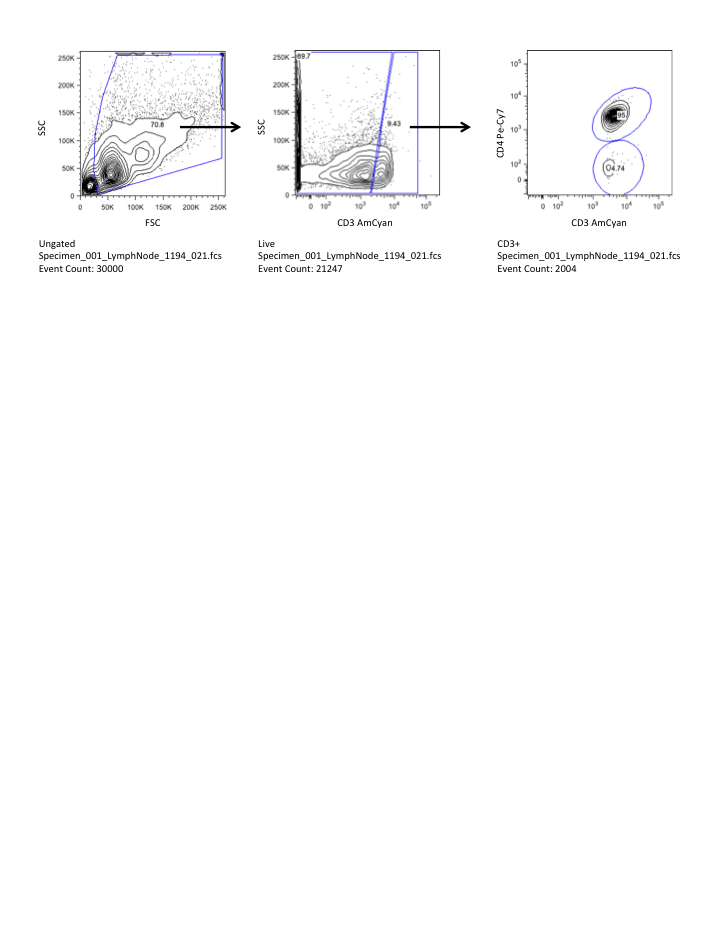


Figure S2: Gating scheme for flow analysis.
